# Community Pharmacists’ Knowledge, Attitudes, and Readiness to Provide Counseling on Food Supplements—A Scoping Review

**DOI:** 10.3390/nu17233754

**Published:** 2025-11-29

**Authors:** Katerina Slavcheva, Radiana Staynova, Nelina Neycheva, Daniela Kafalova

**Affiliations:** Department of Organisation and Economics of Pharmacy, Faculty of Pharmacy, Medical University of Plovdiv, 4002 Plovdiv, Bulgaria; katerina.slavcheva@mu-plovdiv.bg (K.S.); nelina.neycheva@mu-plovdiv.bg (N.N.); daniela.kafalova@mu-plovdiv.bg (D.K.)

**Keywords:** food supplements, community pharmacy, pharmacist, knowledge, nutrition, safety

## Abstract

Food supplements (FSs) are widely used by the general population and are commonly available in community pharmacies. As highly accessible healthcare professionals, pharmacists are well positioned to provide evidence-based information and guidance regarding their safe and appropriate use. Adequate knowledge of FSs is essential for pharmacists to prevent adverse effects, identify potential interactions with other medications, and ensure rational use. The objective of this study was to assess community pharmacists’ knowledge regarding FSs and their attitudes towards dispensing and patient counseling practices. A literature review was carried out using the scientific databases PubMed, Scopus, and Web of Science. The following keywords were used: (“food supplements” OR “dietary supplements”) AND (“pharmacists’ knowledge”) AND (“pharmacists’ attitudes”). A total of 789 articles were identified from the electronic databases, of which 31 met the inclusion criteria. The majority of studies were conducted in Asia, with fewer in Europe, North America, and Australia. Cross-sectional survey-based studies represented the predominant research design. The analyzed studies showed that community pharmacists generally demonstrate insufficient knowledge regarding FSs. Nonetheless, they tend to hold a positive attitude toward the use of FSs and recognize their responsibility to counsel patients on safe consumption. Several barriers affecting pharmacists’ ability to deliver evidence-based guidance were identified, including limited training, lack of basic nutrition education, and insufficient access to reliable information sources. The findings indicate the need for targeted strategies to enhance pharmacists’ competencies and improve the quality of patient counseling in this domain.

## 1. Introduction

Globally, the use and popularity of food supplements (FS) have grown [1]. Based on recent data, the global FS market was valued at approximately USD 192.65 billion in 2024 and is projected to experience substantial growth over the next decade, reaching an estimated USD 414.52 billion by 2033 [2].

According to the European Food Safety Authority (EFSA) FSs are defined as concentrated sources of nutrients (i.e., mineral and vitamins) or other substances with a nutritional or physiological effect that are marketed in “dose” form (e.g., pills, tablets, capsules, and liquids in measured doses) [3]. In addition to vitamins and minerals, other constituents in the FSs may include amino acids, essential fatty acids, fiber, and various plant and herbal extracts [3]. Although FSs may contribute to overall nutrient intake, they are neither designed nor suitable for disease prevention or treatment and should not replace a nutritionally adequate and balanced diet [3].

The ingredients permitted and the product categories included under the term “Food supplement” differ greatly among countries and are identified by various names. For instance, the term “food supplement” is used in the European Union, whereas in the United States (USA) the term “dietary supplement” is legally defined. Such terminological differences may cause the same product to be classified under distinct regulatory categories, leading to substantial variations in regulatory decisions among countries [4].

In the USA, “dietary supplement” is defined in the Dietary Supplement Health and Education Act (DSHEA) and the term includes vitamins and minerals; herbs and other botanicals; amino acids; “dietary substances” that are part of the food supply, such as enzymes and live microbials (commonly referred to as “probiotics”); and concentrates, metabolites, constituents, extracts, or combinations of any dietary ingredient from the preceding categories [5]. Dietary supplements (DSs) are regulated by the Food and Drug Administration (FDA) through DSHEA [6]. In the USA, a premarket approval for DSs is not required, but the manufacturer must guarantee that the products are safe, and the conditions of use are specified on the label [4]. Federal legislation provides a baseline for regulation of the supplement market, but individual states have the authority to adopt and implement their own regulations [7]. In both the EU and the USA, FSs and DSs do not require a formal authorization by a competent authority, as for medicinal products prior to their entry into the marketplace [8].

FSs are also considered a part of complementary and alternative medicine (CAM). CAM consists of a group of medical and health care products that are not included in conventional medicine [9]. A few of the factors driving CAM use are consumers’ discontent with conventional medicine, their expectation of CAM benefits, and their belief that these products are safe. Easy access and social networks are also noted as influencing factors [10].

According to findings from a consumer survey conducted in Europe, almost nine in ten respondents reported having used an FS at least once in their lifetime, with the vast majority of these individuals (93%) having consumed FSs within the past 12 months. Among the FSs most commonly consumed by these respondents during the previous 12 months were vitamin D, vitamin C, and magnesium [11]. Individuals consume FSs for various reasons, including the maintenance of health and well-being, supplementation of nutrient deficiencies not adequately met through diet, and the enhancement of cognitive and physical performance [12,13]. However, using FSs improperly may negatively affect consumers, especially given the uncontrolled information around them [14]. Moreover, FSs in combination with different medications, may lead to adverse interactions, which can be prevented with a correctly provided professional consultation [15].

It is necessary to take both individual and systemic steps to protect consumers from the health risks that come with FSs. In order to make sure that all risk-related clinical conditions, interactions, and adverse effects are considered, patients should receive appropriate and individual counseling [14]. When it comes to patient education, community pharmacists are the most accessible healthcare professionals who could provide information about FSs [16,17,18]. Pharmacists play a crucial role in self-care management by providing valuable information to ensure that consumers receive appropriate health solutions for their specific conditions [19]. The concept of self-care encompasses self-medication and the rational use of non-prescription medicines, FSs, and CAM by patients, as well as other health products, along with lifestyle recommendations such as promoting physical activity [20]. Pharmacists are well positioned to support the community in adopting healthy and positive lifestyle changes and therefore must have basic nutrition knowledge, including a comprehensive understanding of nutrition principles and their practical implications [21]. However, several studies have reported concerns regarding the lack of nutrition knowledge and confidence among practicing pharmacists and pharmacy students [22,23,24,25]. Nevertheless, considerable variability exists in the nutrition education offered by pharmacy schools worldwide, accompanied by a widespread perception that the current training is insufficient [25].

Given that pharmacists regularly advise patients on FSs for a variety of health conditions [16], it is essential that they possess a reasonable level of product knowledge. Despite this requirement, numerous studies have revealed that pharmacists lack confidence in the safety and effectiveness of FSs, as well as interactions between medications and FSs [26,27]. Community pharmacists need additional information, and more training related to FSs, including basic nutrition knowledge, which should be part of every national policy [28].

This study aimed to evaluate the scientific evidence regarding community pharmacists’ level of knowledge regarding FSs, as well as their practice and attitudes toward dispensing and providing counseling to consumers.

## 2. Materials and Methods

### 2.1. Search Strategy and Selection Criteria

**Figure 1:**
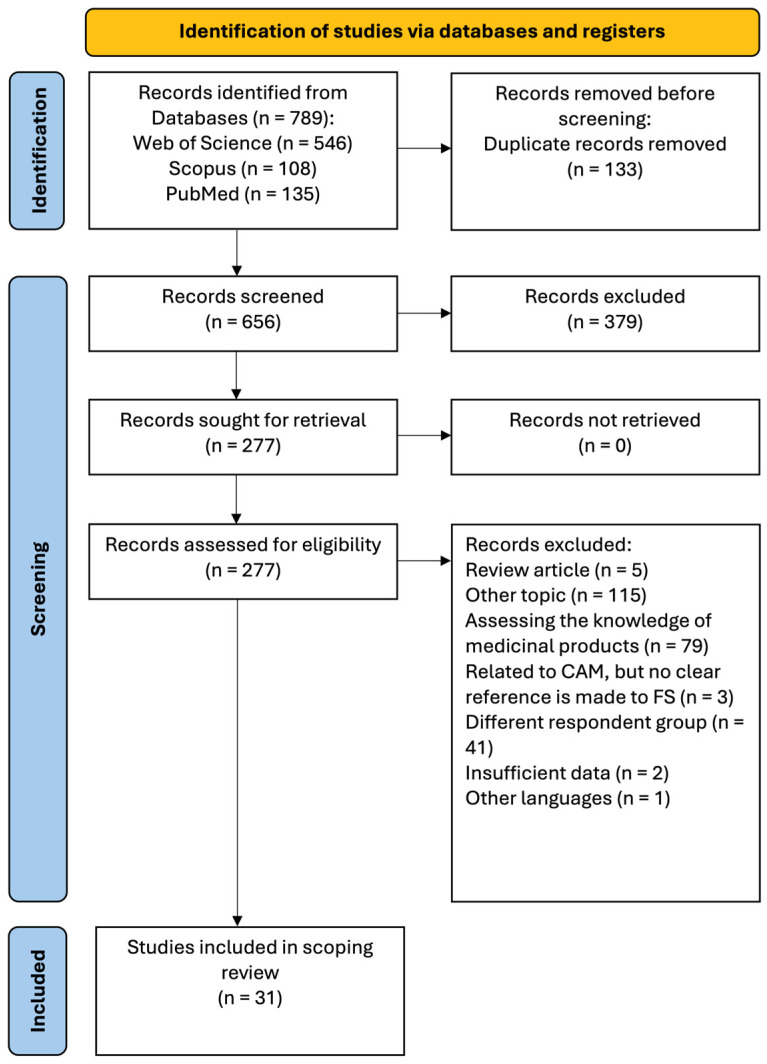
PRISMA flowchart for study selection.

A systematic search was conducted on the PubMed, Web of Science, and Scopus databases. The following keywords were used: (“food supplements” OR dietary supplements”) AND (“pharmacists’ knowledge”) AND (“pharmacists’ attitudes”). The initial search for relevant articles started on 1 August 2025 and was finalized on 26 October 2025. Only studies published between 2000 and 2025 were included. The current scoping review was carried out in accordance with the Preferred Reporting for Systematic Reviews and Meta-Analyses (PRISMA) guidelines (Figure 1) [29].

### 2.2. Eligibility Criteria

The inclusion criteria were full-text peer-reviewed articles written in English that investigated community pharmacists’ knowledge and attitudes regarding FSs. Non-English-language articles and those that did not report on the primary outcome of the current study were excluded from the analysis.

This review did not include editorials, letters to the editor, comments, case reports, narrative reviews, systematic reviews, meta-analyses, or conference abstracts. The eligibility criteria of articles based on the PICOS framework (population, intervention, comparison, and outcome) are presented in Table 1.

### 2.3. Data Extraction

The initial search in the selected databases was carried out by one author (K.S.). In the next stage, duplicates were removed using Microsoft Excel. To remove studies that were deemed ineligible, two authors (K.S. and R.S.) independently reviewed the titles and abstracts of the identified articles. The full text of papers that met the inclusion criteria was retrieved and assessed by two authors (K.S. and N.N.) and rechecked by a third author (R.S.). In case of any discrepancies, they were addressed following a discussion with a fourth author (D.K.).

The following relevant data from each publication that met the inclusion criteria were extracted:

1. Primary author and year of publication,
2. Country,
3. Study design,
4. Sample size,
5. Objective,
6. Study outcomes and main results.

## 3. Results

Using the search strategy, a total of 789 articles were identified from the electronic databases, of which 31 met the inclusion criteria (Table 2). After the removal of duplicates, the remaining 656 papers were screened by title and abstract. Following the screening process, 379 records were excluded due to their irrelevance to the main topic of this review. The full texts of the remaining articles were assessed for eligibility. Of those, 277 were removed for several reasons, as illustrated in Figure 1.

### 3.1. Characteristics of Studies Included in the Review

**Figure 2:**
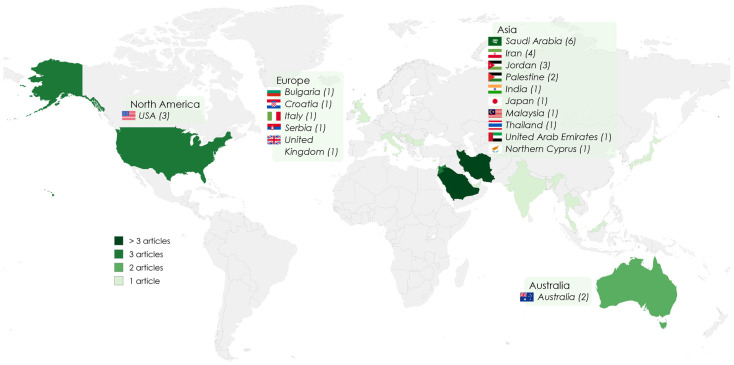
Geographical distribution of studies (Created with MapChart and MS PowerPoint).

Of the 31 articles included in the review, the majority were cross-sectional studies (*n* = 24) conducted using questionnaires administered face-to-face, on site at the pharmacy, online, and postal [14,15,16,17,30,31,32,33,34,35,36,37,38,39,40,41,42,43,44,45,46,47,48,49]. Qualitative studies using interviews (*n* = 5) [50,51,52,53,54], one cross-sectional study using a simulated patient method (*n* = 1) [55], and one mixed-method study using a self-completion questionnaire and semi-structured interviews (*n* = 1) [56] were also included in the review. The studies were published between 2003 and 2025 and originated from 17 countries. Most studies were conducted in Asia (six in Saudi Arabia; four in Iran; three in Jordan; two in Palestine; one each in India, Japan, Malaysia, Thailand, the United Arab Emirates, and Northern Cyprus) compared to fewer in Europe (one in Bulgaria, Croatia, Italy, Serbia, and the United Kingdom), three in the USA, and two in Australia (Figure 2).

Most studies’ participants consisted of only community pharmacists (*n*  =  24). The sample size of community pharmacists in each study ranged from four [40] to one-thousand forty-one [38]. Some of the included studies also enrolled additional participant groups, such as physicians, nurses, dietitians, key stakeholders, general public, intern pharmacists, pharmacy students, pharmacy technicians, and hospital pharmacists [30,36,45,48,52,53,56]. However, in alignment with the objective of the present review, only findings related to community pharmacists holding a university degree were considered.

Although many terms were used to describe FSs, the most common included dietary supplements (*n * =  9), vitamin supplements (*n*  =  5), herbal supplements (*n* = 5), CAM (*n* = 2), nutritional supplements (*n* = 2) and health products (*n* = 1). Some articles specifically focused on weight reduction supplements and herbal products (*n* = 1), folic acid (*n* = 2), iodine supplementation (*n* = 1), melatonin supplements (*n* = 2), probiotics (*n* = 1), and vitamin D (*n* = 1). The details associated with all the eligible articles’ characteristics can be found in Table 2.

**Table 2:** Characteristics of included studies.

Authors, Year	Country	Study Design	Sample Size and Participants	Objective	Study Outcomes and Main Results
Kemper et al., 2003 [30]	The United States of America	Cross-sectional questionnaire-based study	*n* = 537 - physicians (*n* = 111) - nurses (*n* = 30), - pharmacists (*n* = 46), - dietitians (*n* = 350)	To investigate healthcare professionals’ (HCPs) knowledge, attitudes, and practices related to herbs (H) and FSs.	Although participants showed interest and prior training in H/FSs, their knowledge, attitudes, and practices remained suboptimal, highlighting the need for further education and supportive institutional policies.
Shilbayeh, 2011 [31]	Jordan	Cross-sectional questionnaire-based study	*n* = 388 community pharmacists	To give a platform for analyzing the professional practices, attitudes, and knowledge of Jordanian community pharmacists in providing patients with safe vitamin consumption advice.	Community pharmacists were sufficiently aware of their role in counseling.
Robinson et al., 2011 [50]	The United Kingdom	Qualitative study using interviews	*n* = 4 community pharmacists	To investigate attitudes and behavior of community pharmacists regarding children’s use of herbal and nutritional products.	Pharmacists seem to understand the customer’s need for herbal and nutritional products for children. However, pharmacists may require additional information to maintain their professionalism.
Asahina et al., 2012 [54]	Japan	Qualitative study using focus group interviews	*n* = 16 community pharmacists	To investigate how Japanese community pharmacists perceive patients’ use of herbal and dietary supplements.	A number of participants voiced opinions supporting their use, while others disagreed. Some participants agreed as long as it was for preventative purposes. The safety of health products and the lack of scientific proof for their effectiveness were the main arguments against use.
Mehralian et al., 2014 [32]	Iran	Cross-sectional questionnaire-based study	*n* = 500 community pharmacists	To assess the knowledge and measure the professional attitude and practice of pharmacists about FSs.	Although various factors, such as pharmacy ownership, educational background, and professional experience, influence pharmacy practice in relation to FSs, pharmacists’ knowledge should be recognized as the principal determinant. Accordingly, more emphasis should be placed on enhancing FS-related education and training to improve professional practice.
El-Mani et al., 2014 [33]	Australia	Cross-sectional questionnaire-based study	*n* = 41 community pharmacists	To assess pharmacists’ knowledge about folic acid and iodine supplementation during pregnancy and to conduct an audit of pregnancy supplements available online.	There was little awareness among pharmacists regarding the mandatory fortification program and the dietary sources of iodine and folic acid.
Shraim et al., 2017 [34]	Palestine	Cross-sectional questionnaire-based study	*n* = 281 community pharmacists	To evaluate the knowledge, attitudes, and practices of Palestinian community pharmacists regarding CAM.	Despite having fair to average knowledge scores, pharmacists still require additional CAM education to be better equipped to provide pharmaceutical care and enhance patient outcomes.
Bastani et al., 2017 [35]	South of Iran	Cross-sectional questionnaire-based study	*n* = 200 community pharmacists	To ascertain pharmacists’ performance, attitude, and level of knowledge regarding FSs.	The findings suggested that more knowledgeable pharmacists perform better and have a more positive attitude toward FSs.
Dabaghzadeh et al., 2018 [55]	Iran	Cross-sectional study, using simulated patient method	*n* = 84 community pharmacists	To evaluate the counseling practices of community pharmacists relating to typical use, interactions, contraindications, and adverse effects of multivitamin supplements using simulated patients.	The inadequate pharmacy practices identified in this study concerning multivitamin supplements may be explained by insufficient collection of patient information, inadequate counseling competencies, and communication deficiencies.
Ung et al., 2019 [52]	The United States of America	Qualitative study using in-depth interviews	*n* = 22 - pharmacists (*n* = 12); - key stakeholders (*n* = 10)	To assess pharmacists’ and other key stakeholders’ perceptions and opinions about assuming roles that ensure the appropriate and safe use of FSs.	A significant gap persists between pharmacists’ awareness of FS use and their professional responsibilities in this field, highlighting the need for collaborative strategies to enhance their role in ensuring the safe and appropriate use of FSs.
Harnett et al., 2019 [53]	The United States of America	Qualitative study using in-depth interviews	*n* = 22 pharmacists (*n* = 12) and organizational representatives (*n* = 10)	To gather the views of pharmacists and key stakeholders on what is needed for pharmacists to fulfill their professional role related to the use of FSs by Americans.	Despite existing challenges, pharmacists and stakeholders recognize opportunities to strengthen pharmacists’ professional role and promote the safe and appropriate use of FSs in the USA.
Ghosn et al., 2019 [17]	Saudi Arabia	Cross-sectional questionnaire-based study	*n* = 102 community pharmacists	To evaluate knowledge, attitudes, and practices of Saudi community pharmacists in counseling patients regarding the safe usage of vitamins.	The study revealed a positive attitude of community pharmacists on their role in patients’ counseling about the safe usage of FS, but their level of knowledge about these products needs to be improved to meet consumer’s needs.
Wahab et al., 2019 [51]	Thailand	Qualitative study using interviews	*n* = 22 community pharmacists	To evaluate how pharmacists perceive the use of food and herbal supplements and what factors or underlying beliefs affect community pharmacists’ provision of these products.	Although the pharmacists interviewed agreed that providing pharmacist care to users of herbal and FSs had advantages, there were many obstacles to its implementation.
Zeković et al., 2019 [36]	Serbia	Cross-sectional questionnaire-based study	*n* = 730 valid questionnaires - community pharmacists (*n* = 450), - intern pharmacists (*n* = 74) - pharmacy technicians (*n* = 206)	To evaluate community pharmacists’ and pharmacy technicians’ knowledge, attitudes, and practices (KAP) regarding advising women on the appropriate intake of FSs containing folic acid.	Despite participants’ generally positive attitudes regarding the importance of folic acid for maternal and fetal health and their recognition of their role in promoting preconception health, some knowledge gaps were identified.
Alshahrani SM., 2020 [37]	Saudi Arabia	Cross-sectional questionnaire-based study	*n* = 1041 community pharmacists	To ascertain the Saudi Arabian community pharmacists’ knowledge, attitudes, and practices regarding vitamin and nutritional supplement counseling.	Saudi Arabian community pharmacists are knowledgeable about vitamins and nutritional supplements and have a positive attitude toward them.
Altamimi et al., 2021 [39]	Palestine	Cross-sectional questionnaire-based study	*n* = 194 community pharmacists	To assess community pharmacists’ knowledge, attitudes, and practices regarding FSs.	Participants’ knowledge of FSs was found to be moderate, according to the study. Knowledge and practices were also closely related.
Bukic et al., 2021 [16]	Croatia	Cross-sectional questionnaire-based study	*n* = 102 community pharmacists	To investigate factors that might affect pharmacists’ recommendations of FSs and to compare their perspectives and understanding of these products across pharmacists with varying years of work experience.	On the general knowledge test regarding FS, all of the participating pharmacists received high scores. Senior pharmacists who may have missed that component of their formal education should receive extra instruction on evidence-based pharmacy practice.
Brunelli et al., 2022 [14]	Italy	Cross-sectional questionnaire-based study	*n* = 232 - with degree in pharmacy (*n* = 179) - with degree in chemistry and pharmaceutical technologies (*n* = 53)	To evaluate the FS knowledge, attitudes, and practices of pharmacists working in both public and private licensed pharmacists.	Pharmacists have little understanding of FSs, and young professionals need to keep learning in order to provide patients with safe and useful advice regarding these products.
Jalil et al., 2022 [40]	Jordan	Cross-sectional questionnaire-based study	*n* = 401 community pharmacists	To explore Jordanian community pharmacists’ perspectives and knowledge of herbal supplements available in pharmacies.	The mean knowledge score regarding knowledge of use, regulation, adverse reactions, and drug interactions was 3.7, 3.5, 3.6, and 3.6 (out of 5), respectively. Total knowledge scores significantly differed based on work experience (*p* < 0.05).
Alqahtani et al., 2022 [41]	Saudi Arabia	Cross-sectional questionnaire-based study	*n* = 246 community pharmacists	To assess community pharmacists’ knowledge, attitudes, and practices about vitamin D and other FSs.	Pharmacists demonstrated a highly positive attitude toward vitamin D and other FSs, and their overall knowledge levels were correspondingly high. The study demonstrated a significant correlation between the pharmacists’ attitude and practice and their level of knowledge about vitamin D and other FSs.
Balkanska-Mitkova et al., 2022 [15]	Bulgaria	Cross-sectional questionnaire-based study	*n* = 151 community pharmacists	To examine the tendency of pharmacists to correctly recommend FSs to chronically ill patients in order to extend the pharmaceutical care and prevent adverse effects.	Most pharmacists are willing to recommend FSs and are able to take part in monitoring the usage of them by patients who are chronically ill.
Tobaiqy et al., 2023 [42]	Saudi Arabia	Cross-sectional questionnaire-based study	*n* = 300 community pharmacists	To assess community pharmacists’ knowledge and attitudes toward dispensing melatonin supplements and their perceived safety and effectiveness.	Awareness rates about melatonin supplement pharmacokinetics and pharmacodynamics among community pharmacists were low.
Patil et al., 2024 [43]	India	Cross-sectional questionnaire-based study	*n* = 226 community pharmacists	To assess community pharmacists’ knowledge, awareness, and attitudes regarding the use of vitamin supplements.	Accurate and sufficient awareness, knowledge, and attitude regarding vitamin deficiencies, their effectiveness, the recommended daily allowance, toxicity, and interactions among pharmacists are lacking.
Xin RK et al., 2024 [44]	Malaysia	Cross-sectional study	*n* = 260 community pharmacists	To assess the knowledge, attitudes, and practices of community pharmacists regarding the provision of counseling services on vitamins and FSs.	Although most Malaysian pharmacists have a sufficient understanding of vitamins and FSs, this study revealed knowledge gaps, especially with regard to less common products.
Şendal et al., 2024 [45]	Northern Cyprus	Cross-sectional questionnaire-based study	*n* = 348 - community pharmacists (*n* = 24) - physicians/dieticians (*n* = 21) - public (*n* = 303)	To evaluate the knowledge, attitudes, and practices of community pharmacists, physicians/dieticians, and general public regarding herbal products used for weight control and slimming.	Approximately half of the pharmacists recommended senna and green tea for weight loss. However, a substantial knowledge gap was observed regarding their safe use, potential interactions, and adverse effects.
Naja et al., 2024 [46]	The United Arab Emirates	Cross-sectional questionnaire-based study	*n* = 373 community pharmacists	To evaluate community pharmacists’ knowledge, attitudes, and practices regarding CAM.	The study findings demonstrated that community pharmacists have generally positive attitudes and practices, as well as good knowledge of CAM functions.
Alshahrani SM, 2024 [47]	Saudi Arabia	Cross-sectional questionnaire-based study	*n* = 224 community pharmacists	To evaluate the attitudes and knowledge of community pharmacists working in the Aseer Region of Saudi Arabia with regard to probiotics.	Probiotics were correctly defined by more than half of the participants, and most of them were able to differentiate between the various potentially dangerous bacterial strains.
Bidoki et al., 2025 [48]	Iran	Cross-sectional questionnaire-based study	*n* = 147 - community pharmacists (*n* = 93) - pharmacy students (*n* = 54)	To investigate pharmacists’ knowledge, attitudes, and practice regarding FSs.	Knowledge and practice scores correlated with pharmacists’ age, education, experience, and professional title. Most pharmacists demonstrated a positive attitude toward providing FS counseling.
Al-Shawabkeh et al., 2025 [49]	Jordan	Cross-sectional questionnaire-based study	*n* = 322 community pharmacists	To evaluate community pharmacists’ attitudes toward the distribution, safety, and efficacy of melatonin supplements, as well as existing knowledge gaps, and suggested educational measures to promote safer use.	Community pharmacists in Jordan demonstrated non-optimal knowledge and awareness regarding melatonin supplements, indicating a need for improvement in their dispensing practices.
Elsayed et al., 2025 [56]	Australia	Mixed-methods study (self-completion of questionnaire and semi-structured interviews)	*n* = 107 - community pharmacists (*n* = 73) - hospital pharmacists (*n* = 17) - specialist pharmacy area (*n* = 9) - other (*n* = 8)	To investigate the attitudes and practices of Australian pharmacists regarding nutrition counseling and the provision of advice on vitamin and mineral supplements, including the adequacy of their education and training in this area.	Pharmacists expressed willingness and potential to provide nutrition counseling; however, improved nutrition education is required to improve confidence in this area.

### 3.2. Knowledge and Awareness of Community Pharmacists Regarding FSs

Regarding the effectiveness, efficiency, and adverse effects of FSs, almost half of the community pharmacists felt they lacked sufficient knowledge [17]. Nevertheless, findings from several studies indicated that respondents generally demonstrated a moderate level of knowledge regarding FSs [39,44]. The vast majority of community pharmacists who participated in the survey conducted by Patil et al. were aware of vitamins as FSs [43]. In another study, the mean score in the knowledge assessment covering the signs of vitamin overdose and deficiency was rated as good [17]. In the study conducted by Brunelli et al., only one-third of community pharmacists exceeded the minimum acceptable level of knowledge regarding FSs, with higher scores observed among those with more professional experience [14]. Notably, the potential carcinogenicity associated with multivitamin overuse and the presence of unlabeled ingredients in FSs were identified as areas with significant knowledge gaps [14].

Pharmacists’ knowledge scores varied considerably based on the length of their professional experience in pharmacy practice [32,40]. Evidence from several studies suggests that more professional experience is associated with significantly higher levels of knowledge [14,33]. According to Jalil et al., community pharmacists self-reported having a generally good level of knowledge regarding the applications of herbal supplements, their regulations, potential adverse effects, and interactions. The overall knowledge score was higher for the use of herbal supplements than for their regulation, interactions, and adverse effects. Moreover, the majority of respondents reported not receiving any patient complaints related to herbal supplement use [40].

Jordanian pharmacists showed a basic level of knowledge regarding vitamins, which was insufficient to satisfy customers’ needs [31]. According to another study, despite the widespread use of melatonin, community pharmacists’ knowledge regarding pharmacokinetics, pharmacodynamics, and possible adverse effects was comparatively low [42].

Altamimi et al. identified a significant relationship between community pharmacists’ limited knowledge and their practices concerning FSs [39]. Similarly, Bastani et al. found that pharmacists with higher levels of knowledge demonstrated better professional practices and more positive attitudes toward FSs [35]. Furthermore, another finding suggested that pharmacy ownership is a significant factor influencing and potentially enhancing pharmacists’ practices related to FSs [32].

### 3.3. Attitude and Perceived Role of Community Pharmacists Regarding FSs

According to the studies included in the scoping review, community pharmacists generally hold a positive attitude toward the use of FSs and recognize their responsibility to counsel patients on their safe consumption [17,33,43,44]. Moreover, pharmacists perceive that possessing comprehensive knowledge of FSs is essential for their professional practice and for delivering appropriate patient counseling [16].

In two of the included studies, community pharmacists recognized the significance of their role in promoting preconception health and had a good understanding of the benefits of folic acid supplementation for pregnancy [33,36]. Additionally, they were well-positioned to provide adequate information and advice about appropriate pregnancy supplements [33].

Multiple factors have been shown to influence pharmacists’ decisions to recommend FSs. Personal use represents a primary determinant, followed by professional experience [16]. Furthermore, Bukic et al. reported that community pharmacists with more experience were more likely to suggest FS, which may be attributed to their favorable experiences with these products during practice and the positive feedback received from patients [16]. Moreover, findings from Brunelli et al. indicate that pharmacists’ knowledge level exerts the highest influence on their attitudes and professional practices concerning FSs, with additional contributing factors including degree qualification, professional experience, functional role within the pharmacy, and gender [14].

It has also been observed that patients’ decisions to use FSs are influenced by pharmacists’ advice. This highlights the important role pharmacists play in enhancing patients’ awareness, understanding, and attitudes toward FS use [43].

Many community pharmacists believe FSs enhance overall health and provide economic benefits for pharmacies [43,44]. Only a small proportion of pharmacists reported avoiding FS counseling due to the perceived lack of therapeutic efficacy and commercial motivations [15].

## 4. Discussion

As the use of FSs has grown, more patients are approaching community pharmacists with questions regarding these goods [57]. In this context, we conducted a scoping review to summarize the scientific evidence regarding community pharmacists’ knowledge, attitude, and perceived roles in the selection, recommendation, and dispensing of FSs. A total of 31 research articles from 17 countries were included, highlighting key factors that influence professional practices related to FSs. In addition, the review identifies several barriers that may affect community pharmacists’ ability to provide consumers with evidence-based consultation.

The majority of customers and pharmacists perceive that a pharmacist’s professional responsibilities include recommending effective FSs [52,58]. However, the professional obligations of community pharmacists regarding these products have not been clearly defined, and many do not fully fulfill this role in practice [58]. According to a previously published systematic review, community pharmacists’ knowledge regarding FSs appears to be limited. Considering their professional responsibility to provide accurate health information, pharmacists should offer counseling on FSs with the same level of rigor as for other therapeutic interventions [59].

Findings from a study conducted among Australian pharmacists indicated that the main obstacle preventing community pharmacists from recommending FSs as part of CAM practices was safety concerns, followed by limited knowledge, perceived insufficient evidence, and a lack of time for patient consultations [60]. It is essential for pharmacists to receive additional training in evidence-based pharmacy practice, as this aspect may have been insufficiently emphasized during their formal education [16]. Furthermore, it has been suggested that education on CAM be incorporated into pharmacy curricula to promote the safe and effective use of such products by patients and to enable pharmacists to provide well-informed advice regarding FSs [60].

Community pharmacists should provide patients with evidence-based information to strengthen their role as relevant and trusted healthcare providers [61]. In addition, pharmacists are expected to help patients make informed decisions and be a source of objective information [62,63,64]. According to Brunelli et al., pharmacists were well aware that a healthy lifestyle combined with a balanced diet has a positive effect on nutrient intake and that vitamin deficiency can be associated with various factors [14]. The attitudes and actions of community pharmacists appear to support this, particularly when it comes to advising patients on healthy lifestyles, such as a balanced diet, alcohol intake and smoking cessation, as well as recommendations for natural sources of vitamins. These practices are crucial for maintaining health and well-being and preventing chronic diseases [14].

Effective consultation regarding FSs requires community pharmacists to possess sufficient knowledge of nutrition and recommended intake levels of supplement ingredients. El-Mani et al. reported that pharmacists generally demonstrated satisfactory knowledge of the recommended daily intake of folic acid during pregnancy, whereas their understanding of appropriate iodine intake was substantially lower [33]. This highlights the need to strengthen pharmacists’ knowledge of supplement use in specific physiological conditions, including pregnancy and lactation. Additionally, studies have reported considerable variability in pharmacists’ nutrition-related knowledge and professional practice. For example, a study conducted in Australia found that almost all respondents (98.4%) acknowledged the need to enhance their nutrition knowledge [21]. Likewise, research from Northern Ireland indicated that community pharmacists generally perceive their nutrition education as inadequate, a limitation that is reflected in their professional practice [24]. Additional evidence suggests that most pharmacy students recognize nutrition education as essential and support its integration into pharmacy curricula, with a substantial proportion undertaking supplementary nutrition education via elective courses [65].

Interprofessional cooperation is thought to be crucial for enhancing patient safety, particularly between pharmacists and physicians [66,67]. In order to participate in that collaboration, community pharmacists would require additional training regarding the concepts and practices of evidence-based pharmacy [16].

A contributing factor to community pharmacists not receiving patient complaints related to herbal supplement use could be the fact that these products are increasingly purchased online or from herbal stores rather than pharmacies. Another possible reason may be pharmacists’ lack of training and limited awareness of potential adverse reactions [40].

It is imperative that pharmacists stay up to date on the latest FSs, including vitamins, minerals, and herbs, that are available on the market. To ensure consumers’ safety, community pharmacists should spend additional time to stay familiar with dosage, indications, interactions, contraindications, and adverse effects [44]. In this regard, it is essential for community pharmacists’ level of knowledge about FSs to be improved [17]. Studies show that pharmacists are more likely to have a positive attitude and practice if they possess more knowledge [32]. In addition to knowledge and environment, other factors like pharmacy ownership and experience also have an impact on attitude and practice [32]. There are also some ethical concerns, particularly regarding potential conflicts of interest for pharmacists arising from the profit motive associated with FS sales [14].

In recent years, the FS industry has grown rapidly, often relying on relatively simple production processes with limited oversight of safety, quality, and efficacy due to minimal regulatory requirements [59]. FSs are classified as foodstuffs, making the manufacturer, importer, supplier, or distributor responsible for their safety [3]. Given the global rise in FS consumption, further comprehensive studies are needed to assess their effectiveness and risk profiles [59]. In this regard, enhancing the regulatory framework for the manufacturing, marketing authorization/registration, and post-marketing monitoring of FSs is recommended [14].

**Figure 3:**
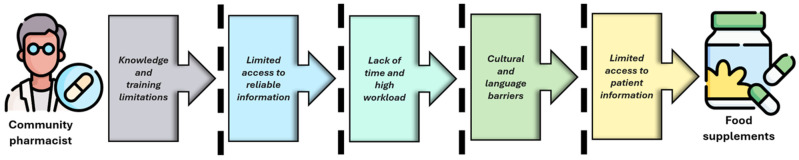
Barriers to the effective practice of community pharmacists in the provision of FSs (Created with MS PowerPoint).

Several barriers may affect community pharmacists’ ability to provide consumers with evidence-based information regarding FSs (Figure 3).

### 4.1. Knowledge and Training Limitations

In addition to the development of pharmacists’ knowledge, education directly affects pharmacists’ attitudes and practices. Therefore, university education and continuous training courses are essential and should receive more attention [32]. In the undergraduate pharmacy core curriculum, more attention regarding formal nutrition education, the pharmacological and toxicological effects of FSs as well as the identification, comprehension, and reporting of adverse reactions should be promoted [25,40].

In order to provide high-quality pharmaceutical care services, concerned bodies must offer community pharmacists access to post-graduation education programs in addition to updating and improving their curricula [17].

Pharmacists should be consistently encouraged to enhance their knowledge and awareness of FSs. To optimize their role in consultation process, pharmacists must be adequately knowledgeable in nutrition principles and their everyday implications [21]. This ongoing professional development enables them to provide more informed guidance to customers and to make evidence-based decisions that are not influenced by commercial considerations such as product availability, pricing, or brand preferences [39].

In this context, several efforts to enhance nutrition education have been undertaken over the past decade. Although it is not required that nutrition courses be part of the basic curriculum, many pharmacy schools offer at least one course [68]. One notable example is the bachelor’s degree program in Nutrition and Dietetics offered at the Faculty of Pharmacy, University of Medicine and Pharmacy, in Cluj-Napoca, Romania [69]. Additionally, postgraduate programs in nutrition are available at multiple universities in Egypt [23]. Similar educational developments have been implemented in Germany, France, the Netherlands, Belgium, the United Kingdom, and the United States [68]. Pharmacy curricula across many European countries integrate a range of topics related to food, nutrition, FSs, and drug–food and drug–FS interactions. In Spain, several universities offer Double Degree programs in Pharmacy and Human Nutrition and Dietetics, designed to provide students with a fundamental knowledge of medicines, food science, nutrition, and dietetics. Institutions offering such programs include the University of the Basque Country (UPV/EHU), the University of Valencia, the University of Navarra, Universidad CEU San Pablo, and the University of Barcelona [70,71,72,73]. Beyond Spain, other European pharmacy schools also incorporate substantial nutrition-related content. For example, the Faculty of Pharmacy at the Medical University of Sofia (Bulgaria) provides a Master-level course in Bromatology for fifth-year students, addressing topics related to basic groups of nutritional substances FSs, and food–drug interactions [74]. Similar bromatology courses are delivered within master’s degree programs at the Faculty of Pharmacy of the University of Porto (Portugal), Alcalá University (Spain), and the Faculty of Pharmacy at the University of Belgrade (Serbia) [75,76,77]. In addition to core curricula, the Department of Bromatology at the University of Belgrade also offers continuing professional development courses for pharmacists on topics such as obesity and dyslipidemia [77]. Furthermore, in Italy, the University of Catania includes a module entitled Physiology of Nutrition within the Degree Course in Pharmacy, emphasizing the role of appropriate nutrition in disease prevention and management while providing students with fundamental nutrition knowledge [78].

### 4.2. Limited Access to Reliable Information

According to Ghosn et al., community pharmacists have adequate general information about FSs, but there is insufficient information regarding potential interactions, indications, and contraindications in special patient populations such as pregnant and breastfeeding women [17]. A substantial proportion of less experienced pharmacists depended on product labeling, even though such labeling offers primarily administration instructions and lacks sufficient information to inform evidence-based decisions regarding the use of FSs [16]. According to a study conducted in Croatia, community pharmacists generally do not rely on credible and up-to-date sources of information about FSs. This pattern was particularly observed among more experienced pharmacists, whereas newly graduated pharmacists were more likely to use data from systematic reviews and clinical trials [16].

### 4.3. Lack of Time and High Workload

Time constraints may limit community pharmacists’ use of high-quality information sources needed to provide necessary counseling related to FSs and contribute to under-reporting of adverse effects, alongside barriers such as limited knowledge, challenges in adverse event reporting, and professional role perceptions [79,80,81]. Senior pharmacists’ administrative responsibilities further reduce their capacity for patient care and evidence-based recommendations on FSs [81].

### 4.4. Cultural and Language Barriers

It should also be emphasized that the presence of language barriers is associated with considerable negative implications for health care services [82]. In the study by Watermeyer and Penn, 2009, pharmacists perceived cultural barriers as more significant obstacles to patient communication than language barriers [83]. High-quality information in a native language could be another barrier for pharmacists in non-English speaking countries [16].

### 4.5. Limited Access to Patient Information

Healthcare services provided by community pharmacists are significantly limited by restricted access to patient health information and inefficient communication channels with prescribers. Integration of electronic health record systems in community pharmacy settings may enhance these processes. However, further research is needed to evaluate the potential benefits and practical implementation [84].

### 4.6. Implications for Practice, Policy, and Future Research

This review provides several recommendations for policy, practice, and future research. The identified barriers compromise good pharmacy practice regarding FSs. These factors should be addressed in order to enable pharmacists to provide patients with adequate counseling regarding the use of these products. The extent of FSs’ problematic use associated with online purchase and monitoring should also be examined. Various strategies are necessary to promote the provision of pharmaceutical care for FS users, including training programs to enhance pharmacists’ confidence, initiatives to encourage the delivery of FS-related pharmaceutical care, and the availability of reliable information resources within community pharmacies [51]. Educational programs should not be relied upon as the primary source of information, despite the tendency of many experienced pharmacists to do so. These programs are typically not subjected to peer review and may not have the critical evaluation required; so, in many cases they might not be the best available evidence on a given topic [16]. On the other hand, community pharmacists should incorporate FS training into their continued professional development, because this can provide them with the most up-to-date and relevant information regarding these products [39]. Consequently, these educational programs should only include information that is supported by scientific evidence [16].

Knowledge about the safety and effectiveness of FSs ought to be adequately discussed in the pharmacy curriculum and reinforced by ongoing professional education. As a result, future pharmacists will be better equipped to advise patients about the appropriate and rationale use and offer evidence-based information [40].

### 4.7. Limitations

This scoping review has some limitations that should be noted. First, several studies may show important results but were not included in this review as they focused on CAM practices, and no clear reference was made to FSs. Additionally, various studies were not included in the review as they focused on different respondent groups, such as pharmacy students, the general population, and other healthcare professionals. Second, all studies that met the inclusion criteria were included in this systematic review irrespective of their quality assessment. Furthermore, the current scoping review includes only peer-review articles. Non-refereed articles and grey literature were excluded, which may have led to the omission of relevant and potentially important findings. Lastly, inconsistency was shown across the results of the included studies due to lack of standardization, whether in the terminology used to describe FSs, the type of data collected, and to a lesser extent the type of FSs registered in each country.

## 5. Conclusions

Community pharmacists play a crucial role in patient education regarding FSs. Several factors, including professional experience, workplace resources, pharmacy ownership, time constraints, higher workload, education, and post-graduate training, influence community pharmacists’ practice regarding FSs. Our findings suggest that community pharmacists have a positive attitude toward their role in patients’ advising about the correct usage of FSs, but their level of knowledge needs to be improved to meet consumers’ needs. It is essential for pharmacists to receive additional training regarding evidence-based pharmacy practice. Furthermore, FS education should be integrated into pharmacy curricula to promote safe and effective patient use.

## Figures and Tables

**Table 1:** PICOS criteria for study selection.

Parameter	Description
Population	Community pharmacists (only pharmacists who have a university degree were considered).
Intervention	Assessment of pharmacists’ knowledge, attitudes, and practices related to FSs counseling and dispensing.
Comparison	Differences in pharmacists’ knowledge or attitudes regarding age, sex, education level, additional training, work experience, or region/country.
Outcome	Level of knowledge regarding FSs, attitudes toward FSs counseling and dispensing, perceived barriers, and educational needs.
Study design	Quantitative (e.g., cross-sectional), qualitative, or mixed-methods studies

## Data Availability

Not applicable.

## Abbreviations

The following abbreviations are used in this manuscript:

CAM	Complementary and alternative medicine
DS	Dietary supplement
EFSA	European Food Safety Authority
FS	Food supplement
HCP	Healthcare professionals
